# Mapping Tree Canopy in Urban Environments Using Point Clouds from Airborne Laser Scanning and Street Level Imagery

**DOI:** 10.3390/s22093269

**Published:** 2022-04-24

**Authors:** Francisco Rodríguez-Puerta, Carlos Barrera, Borja García, Fernando Pérez-Rodríguez, Angel M. García-Pedrero

**Affiliations:** 1 EiFAB—iuFOR, Campus Duques de Soria s/n, Universidad de Valladolid, 42004 Soria, Spain; 2 Föra Forest Technologies sll, Campus Duques de Soria s/n, 42004 Soria, Spain; carlos.barrera@fora.es (C.B.); borja.garcia@fora.es (B.G.); fernando.perez@fora.es (F.P.-R.); 3 Department of Computer Architecture and Technology, Universidad Politécnica de Madrid, 28660 Madrid, Spain; angelmario.garcia@upm.es; 4 Center for Biomedical Technology, Universidad Politécnica de Madrid, 28223 Madrid, Spain

**Keywords:** street level imagery, deep learning, LiDAR, urban trees

## Abstract

Resilient cities incorporate a social, ecological, and technological systems perspective through their trees, both in urban and peri-urban forests and linear street trees, and help promote and understand the concept of ecosystem resilience. Urban tree inventories usually involve the collection of field data on the location, genus, species, crown shape and volume, diameter, height, and health status of these trees. In this work, we have developed a multi-stage methodology to update urban tree inventories in a fully automatic way, and we have applied it in the city of Pamplona (Spain). We have compared and combined two of the most common data sources for updating urban tree inventories: Airborne Laser Scanning (ALS) point clouds combined with aerial orthophotographs, and street-level imagery from Google Street View (GSV). Depending on the data source, different methodologies were used to identify the trees. In the first stage, the use of individual tree detection techniques in ALS point clouds was compared with the detection of objects (trees) on street level images using computer vision (CV) techniques. In both cases, a high success rate or recall (number of true positive with respect to all detectable trees) was obtained, where between 85.07% and 86.42% of the trees were well-identified, although many false positives (FPs) or trees that did not exist or that had been confused with other objects were always identified. In order to reduce these errors or FPs, a second stage was designed, where FP debugging was performed through two methodologies: (a) based on the automatic checking of all possible trees with street level images, and (b) through a machine learning binary classification model trained with spectral data from orthophotographs. After this second stage, the recall decreased to about 75% (between 71.43 and 78.18 depending on the procedure used) but most of the false positives were eliminated. The results obtained with both data sources were robust and accurate. We can conclude that the results obtained with the different methodologies are very similar, where the main difference resides in the access to the starting information. While the use of street-level images only allows for the detection of trees growing in trafficable streets and is a source of information that is usually paid for, the use of ALS and aerial orthophotographs allows for the location of trees anywhere in the city, including public and private parks and gardens, and in many countries, these data are freely available.

## 1. Introduction

In September 2015, the United Nations adopted the 2030 Agenda for Sustainable Development, which includes 17 Sustainable Development Goals (SDGs) that are achieved through 169 targets. In this 2030 Agenda, environmental sustainability is a key component that depends on the sustainable management of the earth’s natural resources. On the other hand, it is unclear how the SDG targets relate to urban ecosystems. Maes et al. [1] define what changes in urban ecosystem management are needed and describe how urban ecosystem management can reinforce or undermine action to achieve the 169 Agenda 2030 targets. Resilient cities incorporate a social, ecological, and technological systems perspective through their trees, both in urban and peri-urban forests and linear street trees, and help promote and understand the concept of ecosystem resilience [2,3].

In the public landscape of cities, trees have been used in two main areas. First, they have been used in spaces for public activities, such as recreational areas, pedestrian walkways, and plazas or parks. Secondly, trees have been used as extensions of the private garden, and more often as street trees in front of houses [4]. Street trees are public resources that complement urban forests and provide numerous benefits to people. However, the value of these urban trees to wildlife is not well understood, which is a gap in our knowledge of urban ecosystem conservation [5,6]. One of the most important and most studied effects of urban trees is their ability to sequester carbon and reduce house cooling energy consumption, due to the shade generated by these trees [7,8]. In short, urban trees play an essential role in making our cities more sustainable, livable, and resilient to climate change. To maximize the benefits of urban trees, city managers need to know where these trees are located and how the different species are distributed in our cities [9].

Urban tree inventories usually involve the collection of field data on the location, genus, species, crown shape and volume, diameter, height, and health status of these trees [10]. Nielsen et al. [11] identify four main ways of acquiring and updating urban tree inventories: satellite-based methods, aircraft-supported methods, digital field inventory through photography or laser scanning, and finally, field surveys. On the other hand, the two most current trends for large-area, low-cost urban tree inventories are [12]: first, the use of Convolutional Neural Networks (CNN) for abstract feature and object extraction in imagery [13], and second, the use of increasingly available, low-cost, and detailed street-level imagery [14], such as Google Street View (GSV) imagery. Moreover, Light Detection and Ranging (LiDAR), aerial photography, and multispectral and hyperspectral imaging have become widely used for earth observation and large-scale analysis of forest ecosystems. Such new remote sensing technologies, in conjunction with novel computer vision (CV) algorithms, allow for the semi-automated identification of urban trees and the automatic identification of their main metrics, such as crown width or total height [15,16,17,18,19,20], which can result in being more time-efficient and less costly when compared to field inventory. These methods have already made it possible to analyze forests at different temporal and geographic scales, progressing from the stand level to the plot level and down to the individual tree level [21,22,23]. In that sense, both active and passive remote sensing are robust alternatives for estimating forestry variables and can also be used for urban trees. Optical data is useful for providing spectral information on species and tree condition [16], while active remote sensing technologies, such as Airborne Laser Scanning (ALS), provide very accurate estimation of individual tree height [24], allowing precise canopy height modeling (CHM) and, therefore, individual tree detection (ITD).

Originally, ITD was performed by using photogrammetric point clouds, whereas ALS is now the main technology for the 3D mapping of trees [25]. Hence, numerous methods for individual tree detection developed for optical imagery have been expanded to LiDAR data. Algorithms for ITD can be divided into those using CHM raster data and those using LiDAR point cloud directly [26]. Most of these algorithms for individual tree detection are based on tree canopies representing the highest part of the landscape and therefore find local maxima (LM) of height within the data at individual tree canopies [17,27]. In addition, given some spurious local maxima might be generated by individual tree branches, smoothing filters are often applied to remove them [28]. As a result, the parameterization of LM algorithms is centered on two parameters: a Smoothing Window Size (SWS) and a Tree Window Size (TWS), which defines a fixed boundary within which the algorithm searches for treetops [28]. Following this treetop detection, segmentation is performed to delineate the canopy boundary of individual trees, which is most commonly based on region growth [29,30], watershed delineation [6], or clustering [31,32,33,34]. 

A data source that has recently received much attention from the urban forest research community due to its low cost and global coverage is general street-level imagery, where the best-known service is GSV [14]. In addition to GSV, other digital platforms have launched street-level panoramic imagery products, such as Apple Look Around (some US and international cities), Microsoft Bing StreetSide (US and some European cities), Baidu Total View and Tencent Street View (Chinese cities), Kakao/Daum Road View and Naver Street View (South Korea), and Yandex (Russia and some Eastern European countries), as well as the corporate crowdsourcing platforms KartaView (formerly OpenStreetCam) and Mapillary (acquired by Facebook) [35]. These street-level images have great potential for researchers as a large repository of panoramic images as a source of urban big data [36]. GSV has been used successfully to assess urban trees on streets and highways [14] and even to assess the state of tree health [37]. GSV is a geospatial platform with extensive worldwide coverage that provides standardized, geocoded street-level imagery in different formats and resolutions at a relatively low cost [9]. GSV Street-level imagery is collected through a panoramic camera, which records single snapshots in time covering a 360-degree range of view, spaced every 15 m, meaning that one tree can be seen in multiple images [38]. GSV data can be accessed online through an official API. In addition to street-level data, another interesting source of data, usually freely available on government geospatial portals, are RGB and near-infrared (NIR) orthoimages. There is a strong correlation between RGB and NIR values of tree pixels and certain parameters such as their leaf area, biomass, or phenotypic activity [39] that is usually addressed through spectral signature. This approach can be further improved by merging NIR and RGB data with other sources of information such as ground-level images or ALS data and applying machine learning (ML) algorithms to them [34]. 

Computer vision is the field that deals with the development of techniques that allow computers to evaluate and analyze images or videos (sequences of images). The most common tasks in computer vision of images include object detection and object classification [40,41]. Deep learning (DL) is a subset of machine learning based on neural networks of multiples layers that attempt to emulate how the brain perceives and understands multimodal information. The multiple processing layers of DL methods are able to learn and represent data at multiple levels of abstraction, thus capturing the intricate structures of large-scale data [42]. Advances in the combination of computer vision [43] and deep learning are enabling automation in street-level data collection in urban environments [44]. These computer vision-based algorithms have been applied to assess urban change [45], building types [46], and urban morphology [47]. With respect to urban tree inventory, street-level imagery in combination with CV has been successfully applied in three key areas: (1) urban tree mapping [4,38], (2) quantification of perceived urban canopy cover [5,12,48,49,50], and (3) estimation of shade provision [51,52,53]. In this work, we have combined (and compared) three of the most common data sources: we have used ALS information, RGB and NIR orthomosaics, and GSV street-level imagery. This information has been used to solve the main objective of an urban tree inventory, which is to locate all trees accurately and inexpensively, based on remote data and without the need for field work. The developed method is novel because it compares (and also combines) a methodology based on CV on GSV images, with another methodology based on ITD in ALS data. Finally, in both cases a filtering of the results is performed through a ML algorithm trained with RGB and NIR orthomosaics. 

## 2. Methodology

### 2.1. Study Area and Validation of Results

**Figure 1:**
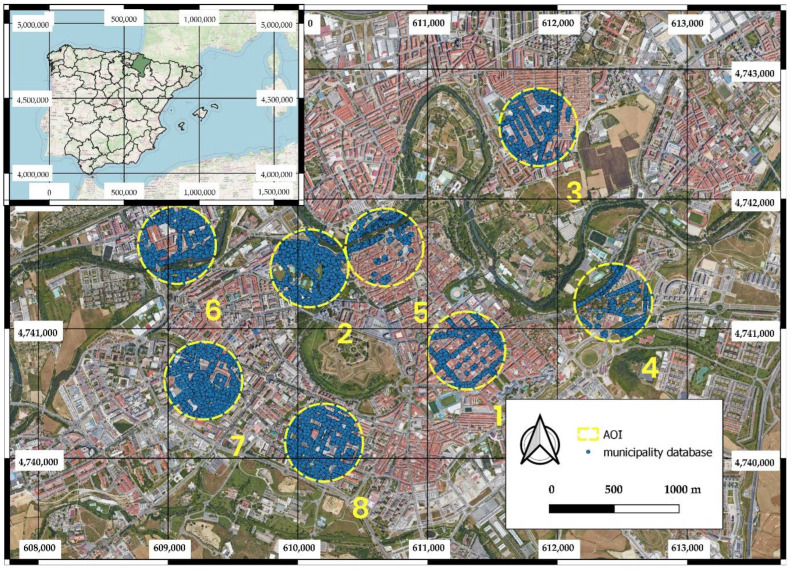
Location of the methodology analysis circles within the city of Pamplona. Circles 1, 2, 3, 6, and 8 were considered fully suitable for vehicles. The rest of the circles (4, 5 and 7) were either pedestrian areas or were residential areas with private gardens.

**Figure 2:**
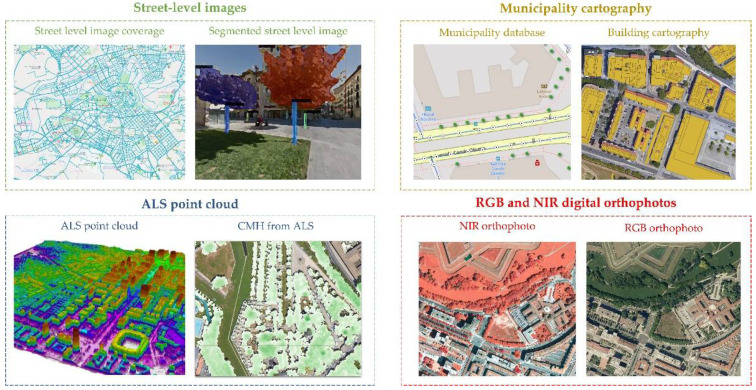
Remote data used in this study.

This work was carried out in the city of Pamplona (Spain). Pamplona is located in northern Spain and is the capital of the region of Navarra (Figure 1). It has a population of 203,944 inhabitants, spread over an area of 25,098 km^2^. It also has 63,962 trees according to its official tree inventory. This city has been selected because it has the three sources of data contrasted in this study: (i) high density ALS data (14 points per square meter), (ii) different complete coverages of GSV images (from 2009 to the present), (iii) several coverages of RGB and NIR orthophotos (from 2005 to the present), and (iv) a complete collection of thematic cartography of the municipality (Figure 2). Another important reason is that Pamplona has a free access database with all the urban trees, where the trees are geolocated with high precision and where their main attributes (genus, species, health status, etc.) are included. This database substantially reduced the field work to collect “ground truth” in the city. Finally, as Pamplona is a large city, it was decided to reduce the study area to different random circles of 300 m radius in which to evaluate the different methodologies proposed. To randomize these eight points, the QGIS software research tool [54] “Vector -> Research tools -> Random points inside polygons” was used to create eight random points separated by a minimum distance of 300 m so that there would be no intersection between their circles. To create the circles, the “Vector -> Geoprocessing -> Buffer” tool of the QGIS software [54] with a radius of 150 m was used. These circles were classified according to three zone typologies: (i) most of the trees are in streets (suitable for vehicles), (ii) most of the trees are in parks (pedestrian only), and (iii) a mix between trees in streets and in parks. Figure 1 shows the 8 selected circles. 

### 2.2. Remote Data Sources

In this work, we have used three data sources: (i) Street-level images, (ii) ALS LiDAR cloud-point data, and (iii) RGB and NIR digital orthophotos. Regarding street-level images, these can be downloaded from Google Street View (GSV), OpenStreetMap, Bing Maps, or we can obtain them ourselves. These images are georeferenced, and we know their heading, pitch, and field of view (FOV). In our case, we have used Google Street View with its corresponding API. Since not all areas were covered at the same time, we had to use images from different years. In total, we used 77 images from 2015, 37 images from 2017, 1100 images from 2018, and 1888 images from 2019. ALS data came from the National Geographic Institute of Spain (IGN). In this case, data were acquired between September and November 2017, with a LEICA SPL100 sensor, obtaining an average point density of 14 first returns per square meter, and with an XY precision of 20 cm and a Z precision of 15 cm. Finally, the orthophoto coverage was also obtained from the IGN. Two coverages were used, one only in RGB carried out in 2014 and another in RGB and NIR captured in 2017. Both coverages provided a pixel size of 0.25 m, a planimetric accuracy in XY of less than 50 cm, an ETRS89 geodetic reference system, and a TIFF file format with its corresponding georeferencing TFW file. In this way, the three data sources were acquired on reasonably similar dates. Figure 2 shows the data sources used in this study.

### 2.3. Geolocation of Urban Trees

**Figure 3:**
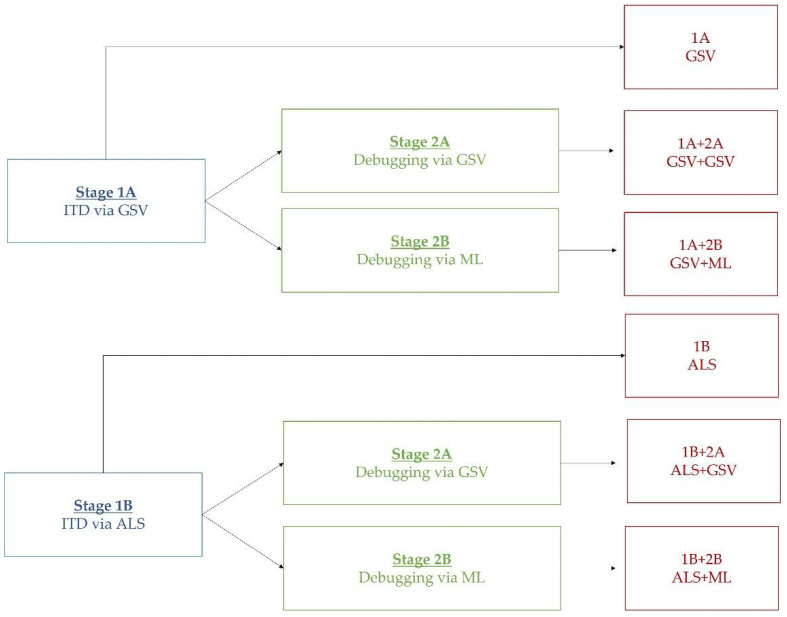
Flowchart of the different stages of the proposed methodology.

Regardless of the database used to geolocate all urban trees, we have designed a methodology based on two stages: (1) detect all possible trees, even knowing that there may be many false positives, and (2) debug those false positives from the previous step. After each stage, we always performed a merging of the trees that were too close to each other (distance less than 4 m), thus eliminating artifacts caused by branches or mispositioning of LiDAR and GSV images. This merging was performed based on the methodology proposed by Picos et al. [55]. In the first stage, two methodologies have been contrasted: (1A) individual tree detection (ITD) through computer vision (CV) on Google Street View (GSV) images, and (1B) ITD from LiDAR point clouds (ALS). In the second stage, two other techniques were again used: (2A) false positive debugging through CV using GSV imagery, and (2B) false positive debugging through ML using RGB and NIR orthophotos. Therefore, four combinations (two in each of the first two stages) were performed and compared to evaluate the accuracy and efficiency of the different results. Results were also included using only the first stage where false positives are not filtered out, as is usual in other investigations consulted [56,57,58,59,60]. It is important to note that the GSV-based techniques are only used to analyze transited street trees (circles 1, 2, 3, 6, and 8), while the LiDAR and orthophoto-based techniques are used to analyze all urban trees. The results were compared with the official data on urban trees, which are freely available on the Pamplona City Council website (https://www.pamplona.es/la-ciudad/geopamplona/descargas, last access on 13 March 2022). Figure 3 shows a scheme of the procedure followed.

#### 2.3.1. ITD through Computer Vision, Using GSV Images (Stage 1A)

**Figure 4:**
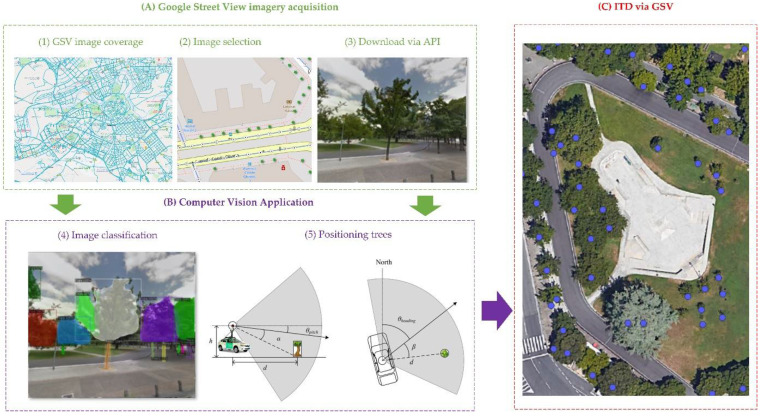
Graphical summary of stage 1A to perform individual tree detection based on the use of computer vision on Google Street View images.

Each image captured from GSV is associated with an identifier called PanoID. Each PanoID has a 360° panoramic image associated with it. These images are georeferenced, and we know their heading, pitch, and field of view (FOV). In two-way streets, images are available in both directions of the street. Google API [61] was used to download the images. The acquisition of GSV images consisted of 3 steps: (1) detecting the areas where GSV images are available, (2) selecting the most suitable images based on date and image parameters, and (3) downloading the images through the GSV API. To detect the presence of trees in each of the images, a model based on MASK R-CNN convolutional neural network (available in the DETECTRON2 library [62]) was retrained. The model was pre-trained with the public database IMAGENET [52]. From this pre-trained model, the model was improved to be able to distinguish between the tree stem and the tree crown. We performed a fine-tuning of the same model using 2000 images manually segmented with LABELME software [53] from other areas of the city of Pamplona. This model was then applied to all the downloaded images and the possible trees were identified. Through the image parameters and based on trigonometric rules we were able to estimate the position of the tree (azimuth and distance to the point where the image was taken). Each detected tree was assigned a unique Id and geolocated based on the azimuth and distance from the origin of the image. Usually, the same tree is detected in more than one image, so its positioning from one or another point should be the same or very close. These possible duplicate trees (if they were less than 4 m away) were eliminated (Section 2.3.5). This methodology, being based on GSV street-level imagery, was only possible on vehicle-traversable streets. Figure 4 shows a schematic of the process followed.

#### 2.3.2. ITD Using ALS Data (Stage 1B)

**Figure 5:**
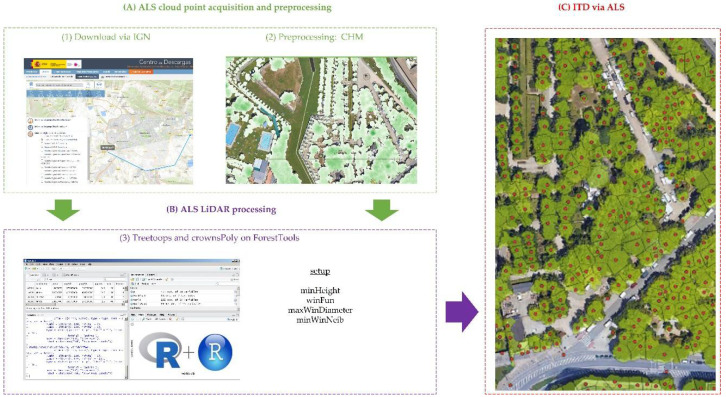
Graphical summary of stage 1B to perform individual tree detection based on the use of ForestTools package on LiDAR point cloud.

Algorithms for ITD can be divided into those that use raster data from the vegetation canopy model (CHM) and those that use the ALS point cloud directly [26]. Most of these algorithms are based on finding local maxima (LM) of height both in the point cloud and in the MDAV [17,27]. Additionally, since individual tree branches can generate some false local maxima, smoothing filters are often applied to remove them [63]. As a result, the parameterization of LM algorithms focuses on two parameters: a smoothing window size (SWS) and a tree window size (TWS), which defines a fixed limit within which the algorithm searches for the tops of the trees [28,64]. Although there are many algorithms to perform ITD, the most common in the forestry field are the packages used from the R software [65], such as FORESTTOOLS [66], LIDR [67] and RLIDAR [68,69], as well as the algorithms integrated in FUSION/LDV [70], such as TREESEG and CANOPYMAXIMA [71]. In this work, a pre-selection was realized and finally the FORESTTOOLS package was chosen, based on a smoothed CHM developed with FUSION [70]. In this way, the LIDAR point cloud data was first downloaded from the IGN website. The smoothed CHM was then generated using FUSION software (CANOPYMODEL procedure, cellsize = 0.25 and smooth = 3). This CHM was clipped with the vector layer of buildings that can be downloaded from the Pamplona City Council website. From this clipped CHM, treetops were found with the R package FORESTTOOLS. Different parameterizations were evaluated, and it was considered that the one that offered the best results was the following: “minHeight = 2; winFun = 0.12 x + 0.5; maxWinDiameter = NULL; minWinNeib = queen”. Each of the treetops had a unique Id and incorporated the height measured over the CHM. Figure 5 shows a schematic of the process followed.

#### 2.3.3. False Positive Debugging through CV Using GSV Imagery (Stage 2A)

**Figure 6:**
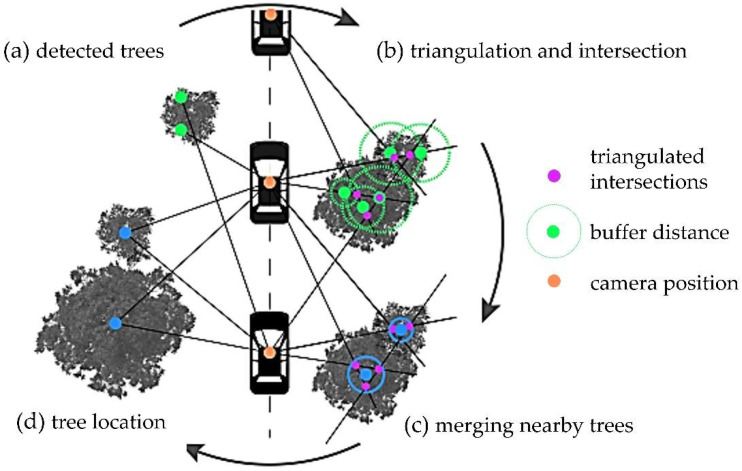
Graphical summary of stage 1A to perform individual tree detection based on the use of computer vision on Google Street View images.

In stage 1A, we selected all GSV images every 10–15 m and we applied the object detection algorithm on all of them, identifying in each of them each tree (stem and crown) with a unique Id. In stage 2A, the procedure was the other way around. First, we started from the trees identified in stage 1, then we selected the three closest images to each of the trees and downloaded them through the Google API. For each of the trees, we analyzed whether that tree was detected in the three closest images. Then, we positioned it using trigonometry and checked if all three positions obtained were less than 4 m away from each other. When this happened, we considered it to be a single tree and positioned it at the centroid of the three positions. Figure 6 shows a schematic of the process followed.

#### 2.3.4. False Positive Debugging through ML Using RGB and NIR Orthophotos (Stage 2B)

For each tree detected in stage 1, a buffer of 50 cm radius was generated and the zonal statistics (mean and standard deviation) of each of the bands of the different orthophotos (RGB-2017, RGB-2014, and NIR-2017) were calculated. A ML algorithm was then trained using a ground truth of 15,766 points sampled from orthophotos to determine whether points detected corresponded to the classes TREE or NOT TREE. To reduce the training processing time, a variable selection was performed using the VSURF procedure [72]. The four most used algorithms in ML for this type of training were evaluated [73]; ANN, SVML, SVMR, and RF, executing the NNET, SVMLINEAR, SVMRADIAL, and RF methods using the CARET package in R software [74]. Finally, a cross-validation was performed using three replicates to control for overfitting. As in the previous method, trees that were less than 4 m away from each other were grouped.

#### 2.3.5. Merging Nearby Trees

The method is based on the methodology proposed by Picos et al. [55] to perform ITD in Eucalyptus. This false positive debugging starts by creating a 2D buffer around the detected and projected treetop. The width of the buffer should be above the X-Y point spacing and below the tree spacing. As the spacing between urban trees is usually larger than in the forest, we tested higher distances than Picos et al. [55], starting at 2 m and ending at 5 m, obtaining the best results for a distance of 4 m. This distance of 4 m coincides with the distance threshold selected by Wegner et al. [38] for considering a tree as a TP. As a result, the point cloud was transformed into a polygon cloud. The intersecting treetops were then combined into a single polygon. These centroids approximate the geospatial position of each individual tree.

#### 2.3.6. Accuracy Evaluation

To validate our results, we have calculated the distance from the suggested point with respect to the closest ground truth point applying the following criteria: (i) if the suggested point is less than 5 m away and there is no other suggested point closer to the ground truth point, we considered it a true positive (TP), (ii) if the suggested point is less than 5 m away from the reference point, but there is another TP point closer, or if the suggested point is more than 5 m away, we considered it a false positive (FP), while (iii) if the ground truth point has no suggested point less than 5 m away, we considered it a false negative (FN).

To explore the influence of the different methods used, an evaluation of the performance in terms of relative error rate was carried out to evaluate the precision of the proposed method three statistics were used: recall (*r*), precision (*p*), and F1 score. These statistics are widely used to assess the detection error of individual trees [63,75,76]. Recall gives us a measure of trees detected and is inversely related to error of omission, precision implies a measure of trees correctly detected and is inversely related to error of commission, and the F1 score allows us to combine precision and recall in a single value through a modification of its mean. The formulation of these statistics is shown below:

(1) r=TP/(TP+FN)

(2) p=TP/(TP+FP)

(3) F1=(2×r×p)/(r+p)

Precision is a great measure when the data are symmetric (similar number of FPs and FNs), and where both errors have the same influence. In our case, the FPs have less influence since in stage 2, debugging, and our goal is to minimize them, so it is better to use F1. On the other hand, recall refers to the number of TPs with respect to all detectable trees (n), and it is the most important statistic when your aim is to have the maximum number of TPs, even if the FPs are also numerous. For this work, we have assessed the trade-off between the three statistics.

## 3. Results

**Figure 7:**
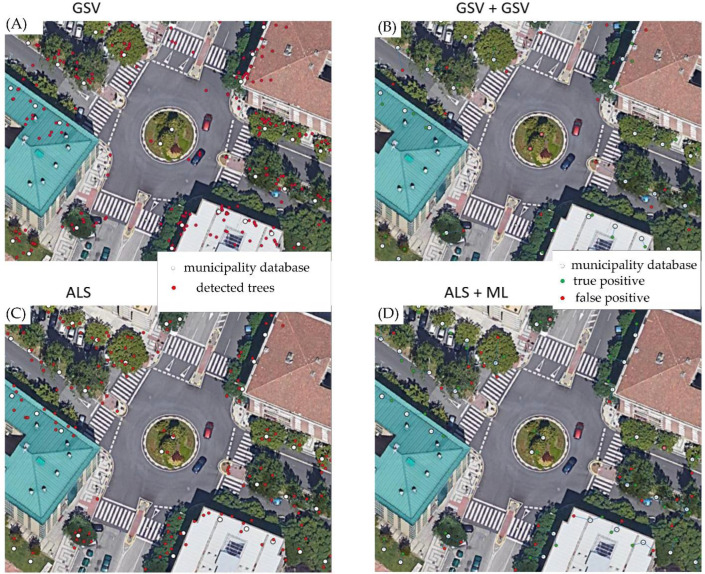
Example of the methodology in one of the areas of the city. (**A**) ITD through GSV imagery and (**C**) ITD through ALS. In this first stage, the white dots indicate the trees from the municipality’s database, while the red dots indicate the trees detected by the methodology. The images on the right show the combination of stages. (**B**) ITD through GSV and FP debugging through GSV, (**D**) ITD through ALS and FP debugging through ML. Green dots indicate well-identified trees (TPs) and red dots indicate incorrectly detected trees (FPs).

Table 1 shows the results obtained in each of the combinations of stages. In order to compare methodologies, we focused only on the areas where GSV imagery is available (areas trafficable by vehicles). The results obtained in the first stage are very satisfactory, identifying more than 86% of urban trees. Once the FP debugging is performed (second stage), recall decreases to 78%, given that this process removes some true positives, while eliminating a large number of FPs. Figure 7 shows an example of four of the six methodologies tested in one of the areas of the city. The left column shows the first stage (ITD through computer vision (A) or ALS (C)), while the right column shows the combination of stages (ITD and false positive debugging).

If we focus on recall, a higher value indicates that we have more true positives (regardless of the false positives we found). We have obtained very similar results in the ITD with both ALS and GSV. Regarding FPs in this first stage, we generally found more when using GSV. Therefore, if we are not conditioned by FPs, we always detect more TPs using only the first stage than by combining stages. While the results obtained with both GSV and ALS are similar, they are slightly higher when using ALS.

Considering the second stage to debug FPs, GSV- and ML-based methods are both equally valid. The combination of all stages is somewhat better when starting from an ITD performed with ALS, although differences are not significant. In general, the best result is obtained by performing the first stage with ALS and the second with GSV. Even so, the advantage of the ALS + ML method is that it works for any place where ALS and orthophoto data exist, which allows the inventory to be performed also in public and private parks and gardens and in pedestrian areas.

## 4. Discussion

The use of street-level imagery through CV techniques has recently been employed for mapping urban trees [4,38]. Berland and Lange [14] used GSV and obtained 93% of recall on urban trees and discovered that it was possible to assess genus, species, location, diameter at breast height, and tree health. Rousselett et al. [37] were capable of identifying trees affected by pine processionary with a 96% success rate. However, these studies and many others were not automated, so they were limited by costly manual effort. In addition, Li et al. [5] estimated a factor to quantify tree shade provision and assessed the percentage of vegetation on streets by measuring the number of green pixels observed in a GSV image. As for Seiferling et al. [48], they quantified urban tree canopy cover using GSV and ML. These methodologies were the origin of the Green Vision Index [5]. Wegner et al. [38] designed a workflow for automatic detection and geolocation of street trees from GSV and Google Maps images, based on the convolutional neural network model Faster R-CNN. They obtain a recall of 0.706 in tree detection, but also perform a Tree species classification with an average recall of 0.79 (varying as a function of the species classified). This study is more complete than ours, since it identifies the species, but it is the most comparable to ours, in terms of methodology and results.

Methodologies based on ALS data for urban tree detection are less abundant but have also been implemented and automated in some major cities. Tanhuanpää et al. [59] were able to detect 88.8% of urban trees using an automated mapping procedure in the city of Helsinki (Finland). They also measured their height, obtaining a Root Mean Squared Error (RMSE) of 1.27 m, and the diameter at breast height (RMSE = 6.9 cm). Holopainen et al. [60] used a non-automated methodology and found that Vehicular LIDAR (VLS) obtained higher recall than ALS (79.22% versus 68.04%, respectively) on a sample of 438 trees located in parks and urban forests of the city of Helsinki (Finland). After automating their methodology, recall dropped significantly for VLS (26.94%) but not so much for ALS (65.53%). Matasci et al. [77] evaluated the urban tree inventory in Vancouver with ALS on a sample of 22,211 trees, obtaining a recall of 76.6%. Furthermore, they estimated their respective heights (RMSE = 2.6 m) and crown diameters (RMSE = 3.85 m) on a subsample of trees. In Munich (Germany), Wu et al. [78] compared VLS and ALS, obtaining a better percentage of detected trees (83.36%) with ALS, compared to VLS (77.2%). Finally, Hanssen et al. [79] performed a comprehensive analysis of urban tree canopy cover in Oslo (Norway) using ALS, obtaining a recall of 73.6%. Finally, although the use of orthophotography is not common in urban tree mapping, some authors report its potential. For instance, Juel et al. [80] combined RGB and NIR orthoimages with data acquired by ALS to train a Random Forest algorithm that was used to map semi-natural coastal vegetation.

In our case, using a fully automated methodology, we located over 86% of the trees (results of the first stage of the methodology, not debugging false positives). After removing false positives during the remaining stages, our recall decreased to 78%, which is comparable to that obtained by other researchers. Moreover, we obtained quite a balanced proportion of TPs, FNs, and FPs, regardless of the data source used during the first stage. We believe that the most important challenge in this study is to achieve a fully automated methodology that allows us to perform an urban tree inventory with the minimum cost of error correction, either through photointerpretation or tree identification at street level. If we included a third phase of photointerpretation in our study, we would certainly be able to clean up almost all the FNs and include almost all the TNs (originally omitted trees).

Furthermore, we have observed that GSV-based methods perform worse in streets where we find different parallel rows of trees, as they cover each other, hence increasing the error rate. This allows for the use of different combinations of methods to perform low-cost and automated urban inventories depending on the available data source (ALS, GSV and/or orthophotos). It should be noted that GSV-based methods only work in areas passable by vehicles, while ALS-based methods work for all areas with a similar error rate, including parks and gardens. In many parts of the world there are open access ALS coverages that can allow for the implementation of this methodology. On the other hand, GSV is available (under license) in almost the whole world. 

Something that has not been evaluated in this work is the measurement of tree crown metrics. If we use ALS data during the first stage of our method, we can segment the tree crown boundary through the usual algorithms [6,29,30,31,32,33], while if we use GSV we can perform tree crown measurements applying semantic segmentation and trigonometry [53,81,82,83]. The difference between the two methods is that when relying on ALS data we can perform measurements on the horizontal axis (crown width), whereas when relying on GSV data we can include measurements from both axes (crown width and crown length).

## 5. Conclusions

This study evaluates, in a cost-effective and accurate way, different methods to detect and geolocate trees in urban environments using several data sources (airborne LiDAR point clouds, street level imagery, and digital orthophotos). In many countries, these sources are freely available, so that all the combinations of methodologies evaluated in this study can be carried out, allowing the inventory of both public and private areas as well as pedestrian areas. Thanks to this inventory, public administrations can have much more precise data on the amount of vegetation in a city, as well as the benefits it generates (e.g., their ability to sequester carbon and reduce house cooling energy consumption, due to the shade generated by these trees).

In this research, we use an innovative approach by prioritizing the detection of the maximum possible number of trees (TPs), even if this also implies a higher number of FPs, since we refine these FPs in a second stage. In these two stages, we combine data and techniques based on object detection, such as the use of street-level imagery, and unsupervised classification data and techniques, which are more common in remote sensing. Although GSV images have been used in this study, any street-level image is valid to replicate the proposed methodology. The same is valid for ALS data, although ALS captured from an aircraft has been used, point clouds captured with unmanned aerial vehicle (UAV) LiDAR systems or even point clouds generated by UAV-photogrammetry integrated with structure from motion (SfM) techniques can be equally valid.

Finally, street-level images can allow us to identify some qualitative (species, genus, health status) and quantitative (height, crown width, etc.) characteristics of trees, and this should be our next challenge, as well as the use of semantic segmentation techniques.

## Figures and Tables

**Table 1:** Results obtained in the urban tree inventory for the different combinations of stages in the circles (only trafficable by vehicles) of the city of Pamplona (n is the official number of trees, TP is the true positives detected, FP is the false positives detected, FN is the false negatives detected, *p* is the precision, *r* is the recall, and F1 is the overall precision). The highest rated combination of methods is identified in bold.

Zone	Method	n	TP	FP	FN	p (%)	r (%)	F1 (%)
**1**	GSV	700	635	911	51	41.07	92.57	56.90
	ALS		608	655	64	48.14	90.48	62.84
	GSV + GSV		581	271	95	68.19	85.95	76.05
	GSV + ML		581	304	95	65.65	85.95	74.44
	ALS + GSV		542	146	141	78.78	79.36	79.07
	ALS + ML		**509**	**51**	**186**	**90.89**	**73.24**	**81.12**
**2**	GSV	215	112	351	73	24.19	60.54	34.57
	ALS		164	205	36	44.44	82.00	57.64
	GSV + GSV		91	104	104	46.67	46.67	46.67
	GSV + ML		98	120	92	44.95	51.58	48.04
	ALS + GSV		102	66	103	60.71	49.76	54.69
	ALS + ML		**141**	**68**	**66**	**67.46**	**68.12**	**67.79**
**3**	GSV	652	461	420	177	52.33	72.26	60.70
	ALS		490	367	132	57.18	78.78	66.26
	GSV + GSV		409	137	227	74.91	64.31	69.20
	GSV + ML		421	142	221	74.78	65.58	69.88
	ALS + GSV		**421**	**87**	**219**	**82.87**	**65.78**	**73.34**
	ALS + ML		393	55	251	87.72	61.02	71.98
**6**	GSV	366	306	345	44	47.00	87.43	61.14
	ALS		341	314	20	52.06	94.46	67.13
	GSV + GSV		282	97	75	74.41	78.99	76.63
	GSV + ML		278	102	77	73.16	78.31	75.65
	ALS + GSV		**308**	**82**	**52**	**78.97**	**85.56**	**82.13**
	ALS + ML		298	76	61	79.68	83.01	81.31
**8**	GSV	839	759	1228	54	38.20	93.36	54.21
	ALS		772	718	50	51.81	93.92	66.78
	GSV + GSV		707	358	105	66.38	87.07	75.33
	GSV + ML		711	389	98	64.64	87.89	74.49
	ALS + GSV		**707**	**270**	**113**	**72.36**	**86.22**	**78.69**
	ALS + ML		612	207	217	74.73	73.82	74.27
**mean zones**	GSV	2.772	2.273	3.255	399	41.12	85.07	55.44
	ALS		2.375	1.541	252	50.99	86.42	64.13
	GSV + GSV		2.070	967	606	68.16	77.35	72.47
	GSV + ML		2.089	1.057	583	66.40	78.18	71.81
	ALS + GSV		**2.080**	**651**	**628**	**76.16**	**76.81**	**76.48**
	ALS + ML		1.953	457	781	81.04	71.43	75.93

## Data Availability

Not applicable.
